# An open-label study to assess the feasibility and tolerability of rilmenidine for the treatment of Huntington’s disease

**DOI:** 10.1007/s00415-017-8647-0

**Published:** 2017-10-26

**Authors:** Benjamin R. Underwood, Zeyn W. Green-Thompson, Peter J. Pugh, Stanley E. Lazic, Sarah L. Mason, Jules Griffin, P. Simon Jones, James B. Rowe, David C. Rubinsztein, Roger A. Barker

**Affiliations:** 1 Fulbourn Hospital, Fulbourn, Cambridge, CB21 5EF UK; 2 Addenbrooke’s Hospital, Hills Road, Cambridge, CB21 2QQ UK; 3 Quantitative Biology, Discovery Sciences, IMED Biotech Unit, AstraZeneca, Cambridge, CB4 0WG UK; 4 Department of Biochemistry, University of Cambridge, Cambridge, CB2 1GA UK; 5 Department of Clinical Neurosciences, and MRC Cognition and Brain Sciences Unit, University of Cambridge, Cambridge Biomedical Campus, Cambridge, CB2 0SZ UK; 6 Cambridge Institute for Medical Research, University of Cambridge, Cambridge Biomedical Campus, Hills Road, Cambridge, CB2 0XY UK; 7 UK Dementia Research Institute, University of Cambridge, Hills Road, Cambridge, CB2 0XY UK; 8 John Van Geest Centre for Brain Repair, Forvie Site, Robinson Way, Cambridge, CB2 0PY UK

**Keywords:** Autophagy, Neurodegenerative disease, Interventional trial, MRI, Cognition

## Abstract

Preclinical data have shown that rilmenidine can regulate autophagy in models of Huntington’s disease (HD), providing a potential route to alter the disease course in patients. Consequently, a 2-year open-label study examining the tolerability and feasibility of rilmenidine in mild-moderate HD was undertaken. 18 non-demented patients with mild to moderate HD took daily doses of 1 mg Rilmenidine for 6 months and 2 mg for a further 18 months followed by a 3-month washout period. The primary outcome was the number of withdrawals and serious adverse events. Secondary outcomes included safety parameters and changes in disease-specific variables, such as motor, cognitive and functional performance, structural MRI and serum metabolomic analysis. 12 patients completed the study; reasons for withdrawal included problems tolerating study procedures (MRI, and venepuncture), depression requiring hospital admission and logistical reasons. Three serious adverse events were recorded, including hospitalisation for depression, but none were thought to be drug-related. Changes in secondary outcomes were analysed as the annual rate of change in the study group. The overall change was comparable to changes seen in recent large observational studies in HD patients, though direct statistical comparisons to these studies were not made. Chronic oral administration of rilmenidine is feasible and well-tolerated and future, larger, placebo-controlled, studies in HD are warranted.

Trial registration: EudraCT number 2009-018119-14.

**Electronic supplementary material:**

The online version of this article (doi:10.1007/s00415-017-8647-0) contains supplementary material, which is available to authorized users.

## Introduction

Huntington’s disease (HD) is a neurodegenerative disorder which presents with a combination of movement, psychiatric and cognitive deficits (reviewed in Bates et al. [1]). It typically progresses with increasing disability to death over the course of 15–20 years. There are currently no disease-modifying treatments available which can alter the progression of HD [2].

From the very first description, it was recognised that the condition is familial, and the underlying genetic mutation, an expanded trinucleotide repeat in the Huntingtin gene on the short arm of chromosome four, was identified in 1993 [3]. This has led to a better understanding of the molecular pathophysiology of the condition, and consequently to rational treatment approaches. One example of this has been the recognition that the mutant protein produced by the genetic mutation is a substrate for degradation by autophagy [4].

Autophagy describes a process where intracytoplasmic vesicles, called autophagosomes, deliver cytoplasmic contents to lysosomes for degradation. It is amenable to up-regulation by drugs in a variety of cellular, fly, zebrafish and mouse models of HD, and this strategy ameliorates signs of the disease in these models. The original drug that was shown to do this was rapamycin, an inhibitor of the mammalian target of rapamycin or mTOR [5]. However, while this drug is approved for human use, it has a significant side effect profile including immunosuppression making it an unattractive option for trialling in patients with chronic neurodegenerative disorders of the CNS. Subsequent drug screens have identified less toxic up-regulators of autophagy which still retain the ability to rescue cells and behaviour in mammalian and other models of HD [6]. One such drug is rilmenidine (*N*-(dicyclopropylmethyl)-4,5-dihydro-2-oxazolamine), an α2 receptor antagonist and an imidazoline I_1_ receptor agonist [7]. It has been extensively used in the clinic as a centrally acting anti-hypertensive agent with no significant side effects compared to placebo at doses of 1 mg per day and with an extensive safety record in human use including elderly subjects. Thus, this agent is an ideal candidate to assess for the feasibility and tolerability of this approach in patients with HD.

Investigating whether such treatments can really slow disease progression in chronic neurodegenerative disorders, such as HD, will require large expensive studies. Indeed, longitudinal observational studies of patients with Huntington’s disease over 2–3 years (for example, COHORT [8] and TRACK-HD [9–12]) have highlighted that definitively studying the effects of disease-modifying agents on HD will require large numbers of patients followed for several years. However, before embarking on studies of this type, it is important to ascertain that the drug to be trialled is well tolerated and the study feasible.

We therefore undertook a first in HD small open-label study over a 2-year period looking at the tolerability and feasibility of rilmenidine as a possible disease-modifying agent. While no account of its effectiveness can be concluded from such a study, it has provided encouraging data which can now be used to plan more definitive large-scale, multi-centre, double-blind, placebo control trials.

## Methods

### Trial design and participants

This was a single centre open-label study carried out with patients recruited from the regional HD clinic at the Cambridge University Hospitals NHS Trust (Addenbrooke’s Hospital), UK. Written informed consent was obtained from all participants.

### Inclusion criteria

1. A confirmed diagnosis of Huntington’s disease on the basis of qualifying clinical signs and symptoms, specifically a Unified Huntington’s Disease Rating Scale 1999 (UHDRS) total motor score of at least 5 [9], and confirmation of a CAG repeat of > 36 in exon 1 of the htt gene.
2. HD stage 2–3 as defined using a UHDRS total functional capacity (TFC) score of greater than 4 [13].
3. Ambulant and able to self-care independently.
4. Aged between 18 and 70.
5. English speaking and able to give written, informed consent.

### Exclusion criteria

1. An ongoing clinically significant and unstable general medical condition [including but not limited to; asthma, chronic obstructive pulmonary disease (COPD), ongoing ischaemic heart disease problems (IHD), congestive cardiac failure (CCF), left bundle branch block (LBBB) or a cerebrovascular accident (CVA)] confirmed via past medical history or baseline medical or physical examination and investigations.
2. Prescribed anti-hypertensive medication or any drug known to be contraindicated or to have an adverse interaction with rilmenidine (viz. a monoamine oxidase inhibitor).
3. Known hypersensitivity to rilmenidine.
4. Ongoing significant mental illness determined by evidence, or a history, of a psychotic or affective (depression or mania) episode in the 6 months prior to baseline Interview as assessed using the Diagnostic and Statistical Manual of Mental Disorders criteria (Fourth Edition with Text Revision; American Psychiatric Association).
5. Prescribed typical or atypical anti-psychotic medication when being explicitly used to treat a psychotic illness (as opposed to the movement disorder of HD).
6. Pregnant or breastfeeding female patients, including those planning to conceive during the period of the trial. Women of childbearing age who were neither pregnant nor planning to conceive during the period of the study were deemed eligible provided they used two forms of contraception, at least one of which had to be a barrier method.
7. Substance (alcohol or illegal/prescription drug) misuse in the 6 months prior to the baseline assessment.
8. Known co-morbid major neurological disorder (including Parkinson’s disease or an established dementia), HIV/AIDS or hepatitis (HBV or HCV).
9. Previous neurosurgery to the brain.
10. Marked clinically adverse abnormalities on laboratory investigations including creatinine clearance < 15 mg/min or creatinine serum level > 177 Umol/l.

### Interventions and outcomes

All patients were given 1 mg rilmenidine daily for 6 months and then 2 mg daily for the next 18 months of the study. Following screening, patients were seen for a baseline assessment and then reviewed at months 1, 3, 6, 9, 12, 18, 24 and 27 (i.e. 3 months after stopping the rilmenidine). Serious adverse events (SAEs) and withdrawals (all primary endpoints) were recorded along with non-serious adverse events, height, weight, vital signs, routine haematology and biochemistry blood measures and ECG occurred as per the schedule of events (Table 1).

**Table 1:** Assessment schedule for participants in the trial

	Baseline	One month review	Months 3,6,9,12,18 and 24	27 months
Medical history, family history and physical examination	X	X	x	X
Weight	X	X	x	X
Vital signs (blood pressure and pulse/heart rate)	X	X	x	X
Haematology	X	X	x	X
Biochemistry	X	X	x	X
12-Lead ECG	X	X	x	X
Review of adverse events	X	X	x	X
Blood sample for metabolomics	X		x	X
Review of concomitant medications	X		x	X
Magnetic resonance imaging (brain)	X			X
Unified Huntington’s Disease Rating Scale	X		x	X
Mini mental State Examination	X		x	X
Neuropsychiatric Inventory	X		x	x
Trail making test	X		x	X
Verbal fluency	X		x	X
Tower of London (One Touch Stockings of Cambridge)	X		x	X
Extra-dimensional/intra-dimensional shifts	X		x	X

Specific measures of the progression of their HD included UHDRS total motor score, functional and independence scales [13] as well as their performance on the trail making test, Mini Mental State Examination (MMSE) [14] and verbal fluency tasks (as assessed by the controlled oral word association test, COWAT).

Other cognitive tests from the Cambridge Automated Neuropsychological Test Automated Battery (CANTAB) [15] were also used and included the mean time taken (latency) and total number of correctly solved problems on the One Touch Stockings of Cambridge, and the total number of errors made prior to and at the extra-dimensional shift stage of the CANTAB intra-dimensional/extra-dimensional set-shifting task (ID/ED) [15]).

The schedule of events is shown in Table 1.

### Sample size and statistical methods

The estimate of the required sample size (*n* = 16) was designed to detect SAEs/AEs rather than a significant change in secondary endpoints and was based on data gathered from our previous studies using metabolomic biomarkers in HD patients [16]. The number of serious adverse events and dropouts was analysed with an exact binomial test, testing whether the rates were greater than an acceptable safety level of 5% per year, or 10% over the course of the study. One-sided *p* values and 95% CI are reported.

For secondary outcomes, the mean rate of progression (slope) was the main parameter of interest. These outcomes were analysed with multilevel models allowing for patient-specific intercepts and slopes. The basic model for the secondary analyses was

$$y_{ij} \sim {\text{Normal}}(\alpha_{{{\text{patient}}[i]}} + \beta_{{{\text{patient}}[i]}} {\text{time}}_{ij} , \, \sigma_{\varepsilon } ),$$

where *y*_*ij*_ is the outcome for patient *i* at time *j*, and *α*_patient_ and *β*_patient_ are the patient-specific intercepts and slopes, respectively. Priors for model parameters were non-informative (or very mildly informative to aid convergence). For positively skewed outcomes, the data were log-transformed and some outcomes used a binomial or negative binomial likelihood if the data was better modelled as proportions or counts. The results are presented as the annual rate of change and the confidence intervals are the 95% highest posterior density intervals. *p* values represent the proportion of the posterior density on the opposite side of zero from the estimated mean. For example, the UHDRS motor score increased by 3.5 units annually, and the associated *p* value of 0.0063 tells us that 0.63% of the posterior distribution had negative values. The data were analysed with the Bayesian software “Stan” and the rstan and rethinking R packages [17, 18].

Given the large number of secondary variables and the lack of direct hypothesis testing, no statistical adjustment has been made for multiple testing.

### Imaging

16 patients underwent structural MR imaging at the Cambridge Biomedical Campus using a 1.5 T GE Medical Systems Discovery MR450 scanner. Structural MRI scans were collected prior to commencement of treatment and at 27 months (i.e. 3 months after treatment ceased) to avoid any effects of the medication on brain volumes and fluid shifts, given its known anti-hypertensive actions. A T1-weighted 3D Bravo fast spoiled gradient echo (SPGR) image was acquired with repetition time (TR) = 8156 ms, echo time (TE) = 3.18 ms, matrix = 256 × 256, in-plane resolution of 1 × 1 mm, 252 slices of 1 mm thickness, inversion time = 900 ms and flip angle = 9°.

Analysis of the MRI data was performed using Freesurfer v. 6 (stable-v6-beta-20151015). The region of interest (ROI) values was imported into SPSS. A paired *t* test was used to estimate the changes in volume between the two time-points.

### Blood sampling and metabolomic analysis

Blood sampling for metabolomic analysis by gas chromatography–mass spectrometry was also undertaken using techniques previously described [16]. Metabolomic analysis is a technique which provides multivariate quantitative analysis of molecular fragments. Measuring the presence of a large number of these fragments and their relative abundance leads to a molecular profile which can help differentiate between disease states and may also be useful as a potential biomarker of progression. There is reason to believe that metabolism is altered in HD and a number of papers have now described metabolomic changes in HD, including from our own group [16]. In this study, we performed sequential longitudinal metabolomic analysis in each patient as an exploratory potential biomarker of disease progression.

## Results

**Fig. 1:**
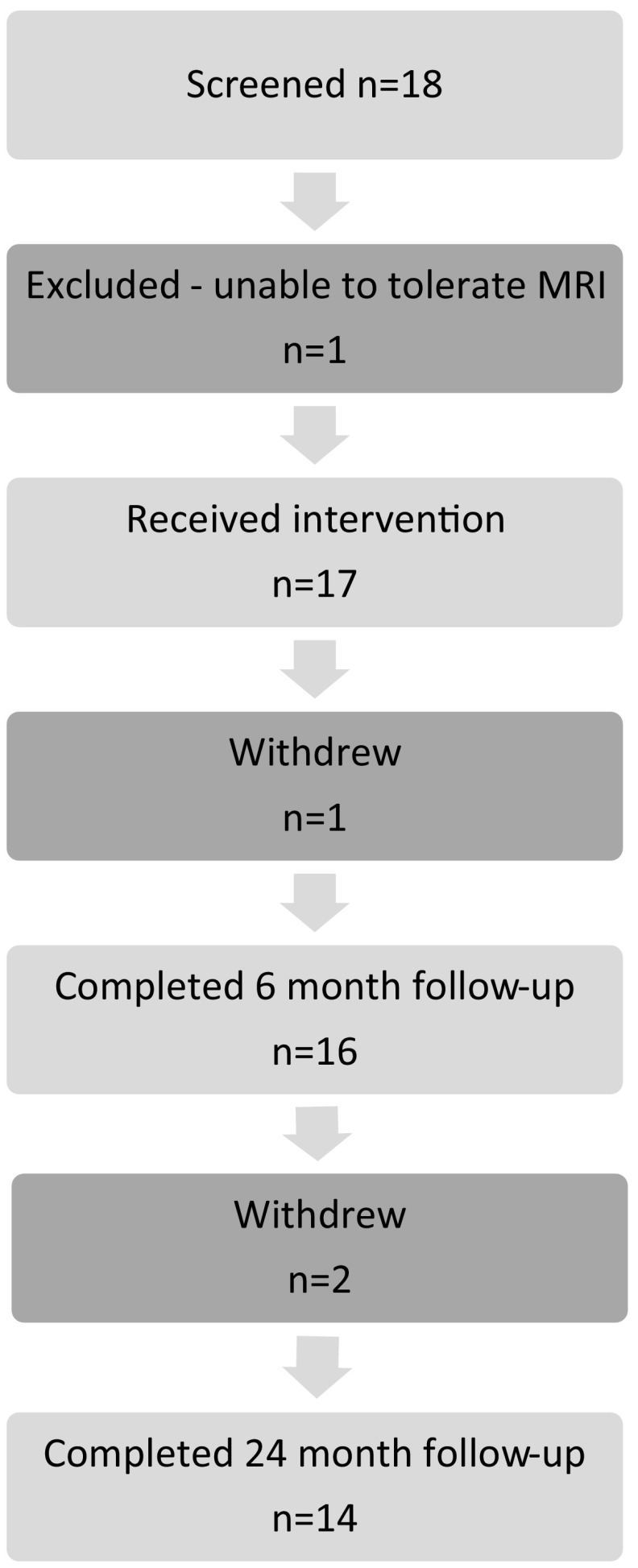
Patient flow through the study

Eighteen patients were recruited to the study. One dropped out before the treatment was started as the patient was unable to tolerate MRI scanning. A further individual dropped out at the 1-month visit as the patient was unable to tolerate venepuncture. This left 16 patients for whom there were more than one observation and who formed the trial population. One patient withdrew from the study after 6 months when they became depressed and required psychiatric admission. One further patient dropped out at 21 months as the patient was no longer able to travel to clinic and two after the 24-month visit, one who no longer wished to continue in the trial and the other was lost to follow-up. Thus, 12 patients completed the 27-month follow-up visit. Excessive movement artefacts in the T1 image of the follow-up visit of one participant meant that 11 patients were included in the longitudinal imaging study. This is summarised in Fig. 1.

### Recruitment

Recruitment occurred between October 2012 and July 2013. The final patient final visit (FPFV) was completed in June 2015.

### Baseline data

Baseline demographic and clinical characteristics are shown in Table 2.

**Table 2:** Baseline demographic and clinical characteristics

Sex	11 male 5 female
Age	Mean 53 (range 37–69)
Weight	80.2 kg (62.5–100)
BMI	27.5 (21.4–33.4)
MMSE	27.4 (24–30)
Verbal fluency	23.2 (6–53)
UHDRS motor	24.5 (7–46)
UHDRS functional capacity	9.62 (5–13)
UHDRS functional assessment	28.2 (25–35)
UHDRS independence	80 (70–100)

*BMI* body mass index, *MMSE* Mini Mental State Exam, *UHDRS* Unified Huntington’s Disease Rating Scale

### Primary outcome measures

Of the 18 patients who were recruited to the study, 6 withdrew for the reasons given above. The number of withdrawals was greater than the pre-specified target rate of 0.1 (*p* = 0.0002, 6/18 patients = 0.33 dropout rate; 95% CI 0.16–1.0). However, none of the withdrawals were considered to be drug-related.

Three serious adverse events (SAEs) were reported: one broken wrist from a physical fall, one admission to hospital for migraine after the individual withdrew their migraine prophylaxis treatment, and one admission for depression following cessation of antidepressants. None were thought to be drug-related.

### Secondary outcome measures

The secondary outcome measures are summarised in Table 3 (secondary outcome measures) and Table 4 (changes in regional brain volume on MRI). Of the secondary endpoints, the UHDRS total motor score (+ 3.5, 95% CI 0.8–6.3, p = 0.009), whole brain volume (− 1.3%, *p* = 0.001) and UHDRS functional capacity (− 0.4, 95% CI 0.8–0.02, *p* = 0.020) showed a decline over the study period as expected.

**Table 3:** Secondary outcome measures (ordered by significance of the *p* value)

Variable	Units	Change per annum	Lower CI	Upper CI	*p* value
UHDRS (motor)	Score	3.5	0.8	6.3	0.009
OTS-latency to correct	Log s	− 0.12	− 0.23	− 0.01	0.014
UHDRS (total functional capacity)	Score	− 0.4	− 0.8	− 0.02	0.020
EDS errors	Number	− 0.2	− 0.5	0.06	0.050
UHDRS (functional assessment).	Score	0.7	− 0.2	1.5	0.063
Trail B	Log s	0.13	− 0.06	0.32	0.081
UHDRS independence scale	Score	− 1.1	− 2.9	0.6	0.100
Weight	kg	0.6	− 0.8	2.1	0.190
Verbal Fluency	Log value	− 0.04	− 0.13	0.06	0.221
MMSE	Score	0.1	− 0.3	0.5	0.263
OTS problems solved on first choice	Log odds	0.06	− 0.18	0.31	0.300
Systolic blood pressure	mmHg	− 0.95	− 5.09	3.16	0.318
Trail A	Log s	0.01	− 0.11	0.13	0.429
Pre ED errors	Log odds	0.05	− 0.70	0.78	0.455

CANTAB One Touch Stockings of Cambridge

*ED* extra-dimensional shift on the CANTAB intra-dimensional/extra-dimensional set-shifting task, *MMSE* Mini Mental State Examination, *UHDRS* Unified Huntington’s Disease Rating Scale

**Table 4:** Changes in regional brain volume on MRI (ordered by the significance of the *p* value)

Region	Percent change	*p* value
Left lateral ventricle	7.2	<0.001
Right lateral ventricle	6.6	<0.001
Brain volume	− 1.3	0.001
Brain volume exc. ventricle	− 1.8	0.001
Left putamen	− 4.4	0.002
Right inferior lateral ventricle	8.1	0.002
Brain stem	− 0.9	0.003
Left cerebellum cortex	− 0.9	0.003
Pons	− 0.8	0.003
Left inferior lateral ventricle	9.6	0.005
Right cerebellum cortex	− 1.0	0.009
Total grey matter volume	− 1.5	0.016
Cerebral white matter volume	− 2.2	0.019
Subcort grey matter volume	− 1.2	0.02
Cerebral cortex volume	− 1.8	0.038
Midbrain	− 0.4	0.174
Right thalamus proper	− 1.4	0.195
Right putamen	− 2.1	0.282
Left amygdala	− 0.7	0.296
Left thalamus proper	− 0.9	0.328
Right amygdala	− 0.6	0.487
Medulla	0.4	0.549
Right hippocampus	− 0.2	0.618
Left caudate	0.8	0.621
Left accumbens area	− 1.2	0.75
Right caudate	1.0	0.797
Right accumbens area	− 0.4	0.905
Left hippocampus	0.0	0.957

Percent volume change per year for selected regions

*p* values from one-sample *t* test

Only one region had a significantly non-normal distribution according to Shapiro–Wilk which was right hippocampus (*p* = 0.04)

In terms of cognitive tests, latency to correct time on the CANTAB One Touch Stockings of Cambridge improved (− 0.12, 95% CI − 0.23 to − 0.01, *p* = 0.014), with no significant changes in any of the other cognitive tests.

The MRI data revealed a decline in total brain volume (excluding ventricles) of − 1.8% per year (*p* = 0.001), although with respect to the basal ganglia, only the left putamen showed a significant decrease in size over the course of the study. Other secondary endpoints did not significantly change over the study.

All other endpoints remained unchanged including the metabolomic analysis where no significant differences were noted between baseline and the end of study including in metabolites that had previously been associated with progression of HD [16]. These complex multivariate results will be reported in a separate paper.

### Haematological, biochemical and ECG changes

Of the routine blood samples taken for haematological, renal and liver function, no consistent or sustained changes were seen. One individual showed a transient rise in creatine kinase which settled by their next visit. Two individuals showed mild and temporary derangement of their LFTs which, on review by a hepatologist, required no further investigation or treatment. All ECGs were reviewed by a consultant cardiologist. Two ECGs showed ventricular ectopics during the study. Follow-up showed no underlying rhythm abnormality and no treatment was required during or after the study. Other AEs are summarised in supplementary Table 2.

## Discussion

This study has shown that it is feasible to undertake a trial of rilmenidine in patients with mild or moderate HD and that rilmenidine was well tolerated. While this 2-year open-label study reported no drug-related serious adverse events and withdrawals, it was also not able to ascertain whether there was any efficacy of this agent as the trial was not designed to test for drug-related changes in secondary outcome measures. However, the data do show changes that are less than, or equal, to that expected in patients at this stage of disease using recent large historical control data [8, 12]. This observation needs to be viewed cautiously given we had no placebo arm in this study and because the large cohort studies of HD populations that have been reported differ in the way they were designed and analysed. However, we did, for example, find a lower rate of generalised brain atrophy (− 1.5% per year seen here versus − 2.05% points per year) and basal ganglia volume loss (+ 0.8 to 1.0% per year seen here versus − 7.46% points per year) than that seen in TRACK-HD [12], a smaller decline in MMSE scores (+ 0.1 points per year seen here versus − 0.7 points per year) [19] and a smaller reduction in the UHDRS total functional capacity score (+ 0.4 points per year seen here versus + 0.6 points per year) than in the COHORT study [20]. In contrast, increases in UHDRS total motor scores were similar (+ 3.5 points per year versus + 3 points per year) to that seen in both the TRACK-HD ([12] and COHORT studies [8]). Weight and cognitive tests did not decline significantly over the course of the study, though it is impossible to rule out the possibility of a learning effect with these cognitive tests.

We also examined metabolomic changes over time, given we have previously found differences in patients with HD and controls [16]. We found no differences over the course of the study. This is the first longitudinal study in HD to investigate metabolomic analysis as a possible study endpoint.

This study has a number of limitations. First, it is open label with a small number of patients. Second, there were several dropouts towards the end of the trial, which although not drug-related, nevertheless reduced the power to draw any conclusions. Third, the study followed patients for only 2 years but effects on disease modification, in either direction, may need longer follow-up to become apparent. Finally, we were unable to measure target engagement in the CNS in terms of whether the rilmenidine truly did up-regulate autophagy at the intended site. Until this can be resolved, studies of this type can only postulate that any effects are mediated via this intracellular pathway.

In summary, this study has shown that rilmenidine appears to be relatively safe and well tolerated in clinically manifest Huntington’s disease. Whether it slows down disease progression through an effect on autophagy is unresolved, but the data from our trial would encourage undertaking further studies with this agent in larger, randomised, placebo-controlled trials.

## Electronic supplementary material

Below is the link to the electronic supplementary material.

Supplementary material 1 (DOCX 15 kb)
