# Supplementary material for: An open-label study to assess the feasibility and tolerability of rilmenidine for the treatment of Huntington’s disease
Source: J Neurol. 2017 Oct 26;264(12):2457–63. doi: 10.1007/s00415-017-8647-0 (PMC5688221; doi:10.1007/s00415-017-8647-0)
Supplement: Supplementary file 1 — Supplementary material 1 (DOCX 15 kb) [file 415_2017_8647_MOESM1_ESM.docx]

**Supplementary Methods- Table 1. Details of the mixed-effects models for each outcome.**

| **Outcome** | **Likelihood** | **Notes** |
| --- | --- | --- |
| Weight | Normal |  |
| Blood pressure | Normal |  |
| Functional assessment | Normal |  |
| Independence scale | Normal |  |
| MMSE | Normal | Max value is 30, with many values at the boundary. Results are only approximate. |
| TFC | Normal |  |
| Trail A | Normal | Data log-transformed. |
| Trail B | Normal | Data log-transformed, 6 censored observations. |
| UHDRS (motor) | Normal |  |
| Verbal fluency | Normal | Data log-transformed. |
| OTS latency | Normal | Data log-transformed. |
| OTS problems solved | Binomial | X solved out of 20. |
| Pre Ed errors | Binomial | X errors out of 50. |
| EDS errors | Negative binomial | Errors as counts, with patient-specific overdispersion. |

Supplementary Table 2 – List of non-serious adverse events

| Headache |
| --- |
| Mild dizziness |
| Diarrhoea |
| Fall in bath |
| Raised serum creatinine kinase on blood test |
| Increased irritability |
| Weight gain |
| Feeling faint |
| Sickness (nausea) |
| Low mood |
| Low mood after Venlafaxine reduced |
| Low WCC and platelet count |
| Injured thumb on a tin |
| Ventricular ectopic on ECG |
| Dry Mouth |
| Rebound sleep deprivation |
| Increased chorea due to Olanzapine stop |
| Asymptomatic sinus bradycardia |
| Asymptomatic sinus bradycardia |
| Recurrence of tingling in the left arm |
| ECG QTc abnormal |
| Pain in left arm |
| Isolated mild raised ALP |
| Sinus tachycardia with ectopics |
| Headache |
| Raised ALT |
| Mild cold |
